# Machine Learning in Dentistry: A Scoping Review

**DOI:** 10.3390/jcm12030937

**Published:** 2023-01-25

**Authors:** Lubaina T. Arsiwala-Scheppach, Akhilanand Chaurasia, Anne Müller, Joachim Krois, Falk Schwendicke

**Affiliations:** 1 Department of Oral Diagnostics, Digital Health and Health Services Research, Charité—Universitätsmedizin Berlin, Corporate Member of Freie Universität Berlin and Humboldt-Universität zu Berlin, 14197 Berlin, Germany; 2 ITU/WHO Focus Group AI on Health, Topic Group Dental Diagnostics and Digital Dentistry, CH-1211 Geneva 20, Switzerland; 3 Department of Oral Medicine and Radiology, King George’s Medical University, Lucknow 226003, India; 4 Pharmacovigilance Institute (Pharmakovigilanz- und Beratungszentrum, PVZ) for Embryotoxicology, Institute of Clinical Pharmacology and Toxicology, Charité—Universitätsmedizin Berlin, 13353 Berlin, Germany

**Keywords:** dental radiography, dentistry, machine learning, neural networks, scoping review

## Abstract

Machine learning (ML) is being increasingly employed in dental research and application. We aimed to systematically compile studies using ML in dentistry and assess their methodological quality, including the risk of bias and reporting standards. We evaluated studies employing ML in dentistry published from 1 January 2015 to 31 May 2021 on MEDLINE, IEEE Xplore, and arXiv. We assessed publication trends and the distribution of ML tasks (classification, object detection, semantic segmentation, instance segmentation, and generation) in different clinical fields. We appraised the risk of bias and adherence to reporting standards, using the QUADAS-2 and TRIPOD checklists, respectively. Out of 183 identified studies, 168 were included, focusing on various ML tasks and employing a broad range of ML models, input data, data sources, strategies to generate reference tests, and performance metrics. Classification tasks were most common. Forty-two different metrics were used to evaluate model performances, with accuracy, sensitivity, precision, and intersection-over-union being the most common. We observed considerable risk of bias and moderate adherence to reporting standards which hampers replication of results. A minimum (core) set of outcome and outcome metrics is necessary to facilitate comparisons across studies.

## 1. Introduction

With the advent of the big data era, machine learning (ML) methods like Support Vector Machine, Naïve Bayesian Classifier, Decision Tree, Random Forest (RF), K-Nearest Neighbor, and Deep Learning involving Convolutional Neural Network (CNN), etc., have been increasingly adopted in fields such as finance, spatial sciences, and speech recognition [1]. Additionally, in medicine and dentistry, ML has been employed for a range of applications, for example, image analysis in dermatology, ophthalmology, or radiology, with accuracy values similar or better than that of experienced clinicians [1,2].

In the field of ML, mathematical models are employed to enable computers to learn inherent structures in data and to use the learned understanding for predicting on new, unseen data [3]. For deep learning models, specifically CNNs, different types of model ‘architecture’ can be used. A ML workflow involves training the model, where a subset of the data is used to learn the underlying statistical patterns in the data, and testing it on a yet unseen, testing data subset. ML models tend to become more accurate, when larger training datasets are used [4]. Moreover, basic learning parameters are usually optimized on a separate data subset, referred to as validation data, a process called hyperparameter tuning. Testing the model on the test data involves a wealth of performance metrics (accuracy, sensitivity also known as recall, specificity, and F-scores, among others), while the assessment of a model’s generalizability, achievable via assessing its performance on an external (independent) dataset, is not frequently performed yet.

Notably, studies in the field of dental ML can vary widely [1]. Different research questions translate into different ML tasks, which in turn necessitate different model specifications. Various input data (numerical, imagery, speech, etc.) can be employed and varied models (SVM, Extreme Learning Machine, Decision Tree, RF, K-Nearest Neighbor, Neural Network, etc.) can be used. Datasets of different sizes and partitions (training, testing, and validation sets) can be used, and a range of methods for balancing the input datasets via synthetic data generation can be conducted. Moreover, the reference test can be established either by having a “hard” ground truth (for example, for imagery, histological sectioning) or fuzzy labeling schemes (for example, multiple human annotators labeling the same image), and a variety of performance metrics can be used to evaluate the model’s performance. These metrics differ with the ML task (classification or, for imagery, detection of objects, or segmentation of specific pixels in an image, or even generation of new images), and can be determined on different hierarchical levels, e.g., patient level, image level, tooth level, surface level or pixel level. Exemplary metrics are accuracy, the confusion matrix and (associated with it) sensitivity (also known as recall), specificity, positive predictive value (precision), and negative predictive value as well as the area-under-the receiver-operating-characteristics curve (c-statistic). For image segmentation tasks (where each pixel has its own classification accuracy), the intersection-over-union (IoU), i.e., the overlap between labeled and predicted pixels (DICE coefficient or Jaccard index), is often used.

As a result, there is significant heterogeneity in the data, tasks, models, and performance metrics, which makes it difficult to contrast studies and assess the robustness and consistency of the emerging body of evidence for ML in dentistry. Additionally, the quality of ML studies—both with regards to the risk of bias but also the reporting of the methods and results—has been shown to vary [5], and with a high likelihood such variance in quality and replicability is also present for dental ML studies.

We aimed to assess this quality of recent ML studies in dentistry, focusing on risk of bias and reporting quality, and to characterize the overall body of evidence with regards to the clinical and ML tasks frequently studied, the model types and underlying datasets, and the employed metrics. Having an overview about these aspects and appraising the consistency and robustness of existing ML studies in our field facilitates to highlight current strengths and weaknesses, and to identify future research needs. In comparison with recent focused reviews on certain clinical tasks (e.g., caries detection on radiographs [6], cephalometric landmark detection [2], etc.), this scoping review not only mainly targets clinical applicability and performance in a subfield of dentistry, but captures the overall picture of ML in our field with a broader focus, and thus a higher number of studies are expected to be included. 

## 2. Materials and Methods

### 2.1. Search Strategy and Selection Criteria

We screened three electronic databases (MEDLINE via PubMed, Institute of Electrical and Electronics Engineers (IEEE) Xplore, and arXiv). Search terms used were ‘deep learning’, ‘artificial intelligence’, ‘machine learning’, ‘convolutional neural network’, ‘dental’ and ‘teeth’. The search strategy for all the three databases used is specified in the Supplementary Materials. No language restrictions were applied. The search was overall designed to account for different publication cultures across disciplines. Reviews, editorials, and technical standards were excluded.

The following inclusion criteria were applied:

(1) Studies which had a dental/oral focus, including technical papers.
(2) Studies employing ML, for example, SVM, RF, Artificial Neural Network, CNN.
(3) Studies published between 1 January 2015 and 31 May 2021, as we aimed to gather recent studies and specifically include deep learning as the most rapidly evolving ML field at present.

Reporting of this scoping review followed the PRISMA checklist [7,8]. Our PICO question was as follows: Which ML practices are being employed by studies in dentistry and what are the methodological quality and findings? The question was constructed according to the Participants Intervention Comparison Outcome and Study (PICOS) strategy.

- Population: All types of data with a dental or oral component.
- Intervention/Comparison: ML techniques applied with a dental or oral focus for the diagnosis, management, prognosis of dental conditions or improving data quality. Patient-level, tooth-level, surface-level, or pixel-level.
- Outcome: Performance evaluation of the ML models in terms of metrics, for example, accuracy, IoU, sensitivity, precision, area under the receiver operating characteristic, F indices, specificity, negative predictive value, rank-N recognition rate, error estimates, correlation coefficients, etc.
- Study design type: For this review, we considered all kinds of studies except reviews, editorials, and technical standards, with no language restrictions.

Ethics approval was not sought because this study was based exclusively on published literature.

Screening of titles or abstracts was performed by one reviewer (A.C.). Inclusion or exclusion was decided by two reviewers in consensus (F.S. and A.C.). All papers which were found to be potentially eligible were assessed in full text against the inclusion criteria. We did not limit the inclusion of studies based on the target study population, outcome of interest, or the context in which ML was used. All original studies related to dentistry and ML, without gross reporting fallacies, such as failure to define the type of ML used, failure to minimally describe which dataset was employed for training and testing, and failure to report study findings, were included in this scoping review.

### 2.2. Data Collection, Items, and Pre-Processing

Data extraction was performed jointly by A.C., A.M., and L.T.A.-S. The extracted data was reviewed by L.T.A.-S. Adjudication in case of any disagreement was performed by discussion (L.T.A.-S. and J.K.). A pretested Excel spreadsheet was used to record the extracted data. Study characteristics included country, year of publication, aim of study and clinical field, type of input data (covariates or imagery [photographs or radiographs; 2-D or 3-D imagery]), dataset source, size and partitions (training, test, validation sets), type of model used and, for deep learning, architecture, augmentation strategies employed, reference test and its definition, comparators (if available, e.g., current standard of care, clinicians, etc.), and performance metrics and their values. In each study, all data items that were compatible with a domain of the extracted data were sought and recorded (e.g., all performance metrics, models employed). No assumptions were made regarding missing or unclear data.

### 2.3. Quality Assessment

The risk of bias was assessed using the QUADAS-2 tool in four domains [9]. First, risk of bias in data selection was assessed using the parameters of ‘inappropriate exclusions’, ‘case-control design’, and ‘consecutive or random patient enrollment’. Second, risk of bias in the index test was assessed using the parameters of ‘assessment independent of reference standard and ‘pre-specification of thresholds used’. Third, risk of bias in the reference standard was assessed using the parameters of ‘validity of reference standard and ‘assessment independent of index test’. Fourth, risk of bias in the flow and timing was assessed using the parameters of ‘appropriate interval between index test and reference standard’, ’use of a reference standard for all patients’, ‘use of the same reference standard for all patients’, and ‘inclusion of all patients in the analysis’. Using the same tool, applicability concerns in three domains were also evaluated. First, applicability concerns for data selection were assessed using the parameter of ‘mismatch between the included patients and the review question’. Second, applicability concerns for the index test were assessed via the parameter of ‘mismatch between the test, its conduct, or its interpretation and the review question’. Last, applicability concerns for the reference standard were assessed via the parameter of ‘mismatch between the target condition as defined by the reference standard and the review question’. We note that alternatively (or even complimentary), the PROBAST tool [10] could have been used for the same assessment.

Adherence to reporting standards was assessed using the Transparent Reporting of a Multivariable Prediction Model for Individual Prognosis or Diagnosis (TRIPOD) tool, which is a 22-item checklist that provides reporting standards for prediction model studies [11]. Note that not all studies included were prediction model studies (studies varied widely in their broader approach, as discussed below), but all involved a mathematical model (ML) for a specific task, which is why we assumed that this checklist would require most studies to adhere to the large majority of domains. TRIPOD has been used for similar purposes in other domains [5]. Risk of bias and adherence to reporting standards were independently assessed by one reviewer (L.T.A.-S.).

### 2.4. Data Synthesis

We describe various aspects of the included studies, such as country of origin, type of input data used, source of datasets, type of ML methods used, etc. We had initially attempted to conduct a meta-analysis using the results of the confusion matrices reported by the included studies; however, out of 168 studies, only 16 (10%) studies presented their confusion matrices in a way that could be used for analysis and furthermore. These studies differed from each other in terms of their clinical research question/task, type of input data, model architecture, etc.

Instead, a narrative synthesis was performed, displaying which ML tasks (i.e., classification, object detection, semantic segmentation, instance segmentation, and generation) have been studied in different clinical fields of dentistry namely, restorative dentistry and endodontics, oral medicine, oral radiology, orthodontics, oral surgery and implantology, periodontology, prosthodontics, and others, i.e., non-specific field or general dentistry. We briefly explain the different tasks in the following section:

- In ML, classification refers to a predictive modeling problem where a class label is predicted for a given example of input data. An example is to classify a given handwritten character as one of the known characters. Algorithms popularly used for classification in the included studies were logistic regression, k-Nearest Neighbors, Decision Trees, Naïve Bayes, RF, Gradient Boosting, etc.
- In object detection tasks, one attempts to identify and locate objects within an image or video. Specifically, object detection draws bounding boxes around the detected objects, which allow to locate the said objects. Given the complexity of handling image data, deep learning based on CNNs, such as Region-based CNN, Fast Region-based CNN, You Only Look Once, Single Shot multiBox Detection, are popularly used for this task.
- In image segmentation tasks, one aims to identify the exact outline of a detected object in an image. There are two types of segmentation tasks: semantic segmentation and instance segmentation. Semantic segmentation classifies each pixel in the image into a particular class. It does not differentiate between different instances of the same object. For example, if there are two cats in an image, semantic segmentation gives the same label, for instance, ‘cat’, to all the pixels of both cats. Instance segmentation differs from this in the sense that it gives a unique label to every instance of a particular object in the image. Thus, in the example of an image containing two cats, each cat would receive a distinct label, for instance, ‘cat1’ and ‘cat2’. Currently, the most popular models for image segmentation are Fully CNNs and their variants like UNet, DeepLab, PointNet, etc.
- A fifth type of a ML task is a generation task, which is not predictive in nature. Such tasks involve the generation of new images from the input images, for example, generation of artifact-free CT images from those containing metal artifacts.

The study protocol was registered after the initial screening stage (PROSPERO registration no. CRD42021288159). 

## 3. Results

### 3.1. Study Selection and Characteristics

**Figure 1:**
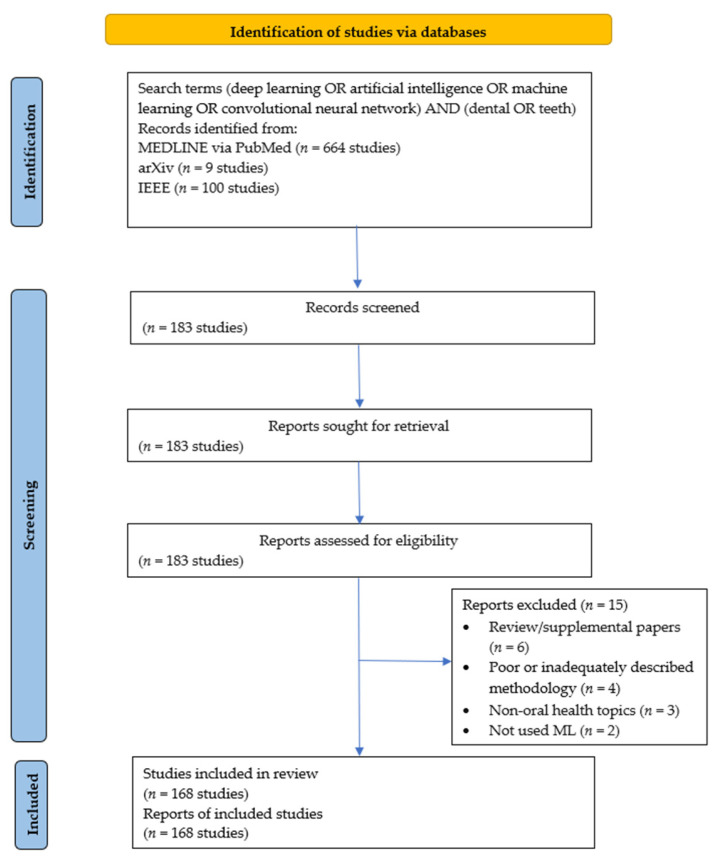
PRISMA study flow diagram.

A total of 183 studies were identified and 168 (92%) studies were included (Figure 1). The included studies [3,4,12,13,14,15,16,17,18,19,20,21,22,23,24,25,26,27,28,29,30,31,32,33,34,35,36,37,38,39,40,41,42,43,44,45,46,47,48,49,50,51,52,53,54,55,56,57,58,59,60,61,62,63,64,65,66,67,68,69,70,71,72,73,74,75,76,77,78,79,80,81,82,83,84,85,86,87,88,89,90,91,92,93,94,95,96,97,98,99,100,101,102,103,104,105,106,107,108,109,110,111,112,113,114,115,116,117,118,119,120,121,122,123,124,125,126,127,128,129,130,131,132,133,134,135,136,137,138,139,140,141,142,143,144,145,146,147,148,149,150,151,152,153,154,155,156,157,158,159,160,161,162,163,164,165,166,167,168,169,170,171,172,173,174,175,176,177] and their characteristics can be found in Table S1. The excluded studies with reasons for exclusion are listed in Table S2. The included studies were published between 1 January 2015 and 31 May 2021 (median: 2019), with the number of published studies increasing each year; 2015: six studies, 2016: four studies, 2017: 13 studies, 2018: 21 studies, 2019: 49 studies, 2020: 68 studies (for 2021, data only until May was available). The included studies stemmed from 40 countries (Figure S1) and used different kinds of input data, such as 2-D data (radiographs: 42% studies, photographs, or other kinds of images: 16% studies), 3-D data (radiographic scans: 18% studies, non-radiographic scans: 4% studies), non-image data (survey data: 10% studies, single nucleotide polymorphism sequences: 1% studies), and combinations of the aforementioned types of data (9% studies). Further, 97% studies used data from universities, hospitals, and private practices, whereas 1% studies each used data from the National Health and Nutrition Examination Survey, M3BE database, 2013 Nationwide Readmissions Database of the USA, and the National Institute of Dental and Craniofacial Research dataset.

Additionally, 85% studies partitioned their total dataset into training and testing data subsets, and 59% studies also created validation data subsets from the same data source. The median size of the training datasets was 450 (range: 12 to 1,296,000 data instances) and of the test datasets was 126 (range: 1 to 144,000). Nearly half of the studies tested model performance on a hold-out test dataset while the remaining used cross-validation. Cross-validation is a resampling method that uses different portions of the data to test and train a model during each iteration. For example, in a 10-fold cross-validation, the original dataset is randomly partitioned into 10 subsamples, out of which nine subsamples are used as training data and one subsample as the test data. Ten iterations of the following step are carried out; the model is trained on the nine subsamples designated as training data and tested on the one subsample of test data; but in each iteration, a different subsample is chosen to serve as the test data and thus a different combination of subsamples constitutes the training data. Eventually, the final estimation of model performance is the average of these results. 

In addition, 65% studies augmented their input data, mainly the training data, but a few augmented the testing data, too. Only 20% studies used an external dataset to validate their model’s performance. The reference test (i.e., how the ground truth was defined) was established by professional experts in 73% studies: one expert in 18% studies, two experts in 11% studies, three experts in 10% studies, four and five experts in 2% studies each, six experts in 1% studies, and seven, eight, 12, and 20 experts in 0.5% studies, each. Another 27% studies used experts for establishing the reference test but did not provide details on the exact numbers. Additionally, 22% studies used information from their datasets as the reference test (for example, age, diagnosis from medical records) and 1% studies used a software tool to generate the reference test. The remaining 4% studies did not provide details on how the reference test was established.

Of all studies, 70% used deep learning models; CNN as classifiers: 59 studies, CNN for other tasks: 14 studies, Faster R-CNN: seven studies, fully CNN: 19 studies, Mask R-CNN: seven studies, 3-D CNN: three studies, adaptive CNN and pulse-coupled CNN: one study each, and non-convolutional deep neural networks: seven studies (Table S1). Another 22% studies used non-deep learning models; perceptron: four studies, other neural networks: three studies, other types of models, such as, fuzzy classifier, SVM, RF, etc.: 30 studies. In addition, 6% studies used various combinations of the aforementioned models and 2% studies did not provide details of the model architecture employed. Both, models using and not using deep learning were employed in higher proportions by studies in restorative dentistry and endodontics, oral medicine, and non-specific field or general dentistry (Table S3). Additionally, models not using deep learning were frequently employed by studies in orthodontics and periodontology. Finally, 20% studies compared their model’s performance with that of human comparators.

### 3.2. Risk of Bias and Applicability Concerns

The risk of bias was assessed in four domains, namely data selection, index test, reference standard, and flow and timing. It was found to be high for 54% of the studies regarding data selection and for 58% of the studies regarding the reference standard (Table 1). On the other hand, the risk of bias was low for the majority of studies regarding the index test (77%) and flow and timing (89%). Applicability concerns were found to be high for 53% of the studies regarding data selection but were low for most studies regarding the index test (79%) and reference standard (73%).

### 3.3. Adherence to Reporting Standards

**Figure 2:**
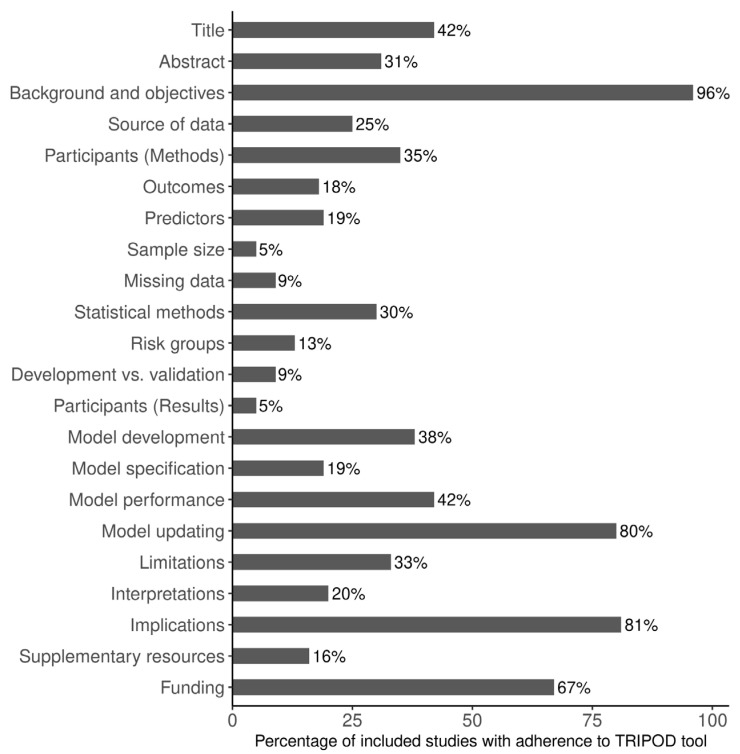
Reporting adherence of studies (*n* = 168) to Transparent Reporting of a Multivariable Prediction Model for Individual Prognosis or Diagnosis (TRIPOD) tool.

Overall adherence to the TRIPOD reporting checklist was 33.3%, with 18/22 domains having an adherence rate less than 50% (Figure 2). Reporting adherence was at or above 80% for background and objectives, and potential clinical use of the model and implications for future research, but below 10% for sample size calculation, handling of missing data, differences between development and validation data, and details on participants. In particular, less than 20% of studies adequately defined their predictors and outcomes (in terms of their blinded assessments), stratification into risk groups, presented the full prediction model and provided information on supplementary resources, such as study protocol, web calculator, or data sets. Less than 40% of the studies adequately reported about their data sources (i.e., study dates), participant eligibility, statistical methods (specifically, details on model refinement), model results (in terms of results from crude models), study limitations, and results with reference to performance in the development data, and any other validation data.

### 3.4. Tasks, Metrics, and Findings of the Studies

Based on the nature of the ML task formulated, the 168 included studies could be classified into five major categories of ML tasks; classification task, *n* = 85; object detection task, *n* = 22; semantic segmentation task, *n* = 37; instance segmentation task, *n* = 19; and generation task, *n* = 5. Classification tasks were most commonly used in oral medicine studies (22%), whereas object detection, semantic segmentation, and instance segmentation tasks, each were most commonly used in non-specific field or general dentistry studies (36%, 38%, and 58%, respectively), Table 2. Generation tasks, though small in number, were most commonly used in oral radiology studies (80%).

A total of 42 different metrics were used by the studies to evaluate model performance and some of these could be grouped into one class, for example, the various correlation coefficients could be combined. Such grouping (or consolidation) resulted in 26 distinct classes of metrics. Note that most studies reported multiple metrics. Studies on classification tasks commonly reported accuracy, sensitivity, area under the receiver-operating characteristic, specificity, and precision, and those on object detection reported on sensitivity, precision, and accuracy. Studies on semantic segmentation reported on IoU and sensitivity, and those on instance segmentation reported on accuracy, sensitivity, and IoU. Lastly, studies using generation tasks commonly reported on peak signal-to-noise ratio, structural similarity index, and relative error. Table S4 shows the number of studies which used the different metrics, stratified by ML task.

After stratifying the studies by ML task and clinical field of dentistry, we attempted to evaluate studies that reported on accuracy, or mean average precision, or IoU. A formal comparison was inhibited by the large variability at the level of clinical or diagnostic tasks amongst the studies.

## 4. Discussion

ML in dentistry is characterized by the availability of a plethora of clinical tasks which necessitate the use of a wide range of input data types, ML models, performance metrics, etc. This has given rise to a large body of evidence with limited comparability. The present scoping review synthesized this evidence and allowed to comprehensively assess this body. We will begin by discussing our findings in detail.

First, the included studies aimed for different ML tasks on a wide variety of data. These data then differed once more within specific subtypes (e.g., imagery, with radiographs, scans, photographs, each of them being sub-classified again, and differing in resolution, contrast, etc.). Moreover, data usually stemmed from single centers, representing only a limited population (and diversity in terms of data generation strategy or technique), all of which likely adversely impacts generalizability of results. The data used were nearly never available, except for the few studies employing data from open databases, leading to difficulties in replication of results. Researchers are urged to comply with journals’ data sharing policies and make their data available upon reasonable request. We acknowledge that there may be data sharing and privacy concerns across institutions and countries. Alternatives to centralized learning of ML models, like federated learning, which do not require data sharing may be of relevance especially for data which are hard to de-identify [178]. Practices of data linkage and triangulation, i.e., using a variety of data sources to create a richer dataset, were almost non-existent. Thus, limiting options for verification of data integrity and increasing the learning output of a ML model by leveraging information from multiple data sources on hierarchical structures and correlations.

Second, a wide range of outcome measures was used by the included studies. These can be measured on different levels, such as patient-level, tooth-level, and surface-level, and while this is relevant for any comparison or synthesis across studies, it was not always reported on what level the outcomes were assessed. Another issue was the high number of performance metrics in use, as evident from our results, leading to only a few studies being comparable to each other. Defining an agreed-upon set of outcome metrics for specific subtasks in ML in dentistry (e.g., classification, detection, segmentation on images) along with standards towards the level of outcome assessment seems warranted. This outcome set should reflect various aspects of performance (e.g., under- and over-detection), consider the impact of prevalence (e.g., predictive values), and attempt to transport not only diagnostic value, but also clinical usefulness. For the latter, studies attempting to assess the value of ML in the hands of clinicians against the current standard of care are needed.

Third, the use of reference tests (i.e., how the ground truth was established) warrants discussion. A wide range of strategies to establish reference tests were employed. In many studies, no details towards the definition of the reference tests were provided. A few studies using image data used only one human annotator as the reference test, a decision which may be criticized given the known wide variability in experts’ annotations [2]. Alternative concepts of applying the reference test to training datasets should be employed and compared to gauge the impact of different approaches and validate the one eventually selected. Additionally, testing datasets should be standardized and heterogeneous to ensure class balance and generalizability. One approach is to establish open benchmarking datasets, as attempted by the ITU/WHO Focus Group on Artificial Intelligence for Health [179].

Fourth, the quality of conducting and reporting ML studies in dentistry remains problematic. Notably, the specific risks emanating from ML and the underlying data are insufficiently addressed, e.g., biases, data leakage, or overfitting of the model. Furthermore, many studies suffered from unclear or a lack of validation of their results on external datasets. The evaluation of a model’s performance on unseen data is a crucial aspect as it relates to the generalizability of ML models regarding performance on data from other sources. Exploration of why some models were not generalizable was even less common, thus preventing identification of steps required to better the models. Generally, the majority of studies performed application testing, developed models, and showed that ML can learn and, in many studies, predict. Understanding why this is, how it could be improved, what the clinical domain needs, or which safeguards for ML in dentistry are required, was seldom an issue. General reporting did not allow full replication, as many details were not presented, and additionally, the display of the model performance remained, as discussed, insufficient. Researchers need to adhere to the published guidelines on study conduct and reporting [180,181,182].

In an effort to characterize the emerging pattern in the included studies, first, we would like to elaborate on the nature of clinical tasks employed by the studies. A wide array of research questions were present; from detecting dental artifacts in images to investigating the benefits of transfer-learning, from classifying different dental conditions to aiding in decision-making and assessing cost-effectiveness. Thus, there is evidence of broadening of avenues where ML could be exploited. As stated earlier, classification tasks were the most common and this may be because diagnosing dental structures or anomalies on images is a vital step towards successful treatment outcomes and prognosis. However, over the years, ML methods have improved their classification performance on images at the cost of increased model complexity and opacity [183]. The inability to explain ML’s methods and decisions is one of the contributing factors towards development of explainable AI, i.e., a set of processes that allows human users to comprehend and trust the results created by ML algorithms. Second, more recent studies tended to employ image segmentation models [2,25,39,48,59,60,73,151].

The presented scoping review has a few salient features. First, it is the most comprehensive overview on ML in dentistry with 168 studies being included. Second, and as a limitation, we could not include randomized controlled trials because none were available and found the included studies to have a considerable risk of bias, both of which should be considered when interpreting our results. Third, to our knowledge this study is the first to employ TRIPOD for gauging the reporting quality of studies using ML in dentistry. TRIPOD is a checklist designed to assess prediction models which has not been validated specifically for ML applications [5]. However, previous studies have used it to evaluate ML models since the quality assessment criteria for clinical prediction tools and ML models are similar [5]. At present, a TRIPOD-ML tool is under-construction [5]. Fourth, we included studies until May 2021 only, as the systematic critique of the 168 studies required considerable time and effort since then. We acknowledge that inclusion of recently published studies may have strengthened our review. Furthermore, we acknowledge that arXiv, an archiving database, may include studies which did not undergo a formal peer-review process and this may be a limitation for our study. However, studies on arXiv are reviewed by peers in a non-formal process and updated after peer-review. Last, any clinical usability cannot be inferred from this study because it was not the focus of this comprehensive review.

## 5. Conclusions

In conclusion, we demonstrated that ML has been employed for a large number of tasks in dentistry, building on a wide range of methods and employing highly heterogeneous reporting metrics. As a result, comparisons across studies or benchmarking of the developed ML models are only possible to a limited extent. A minimum (core) set of defined outcomes and outcome metrics would help to overcome this and facilitate comparisons, whenever appropriate. The overall body of evidence showed considerable risk of bias as well as moderate adherence to reporting standards. Researchers are urged to adhere more closely to reporting standards and plan their studies with even greater scientific rigor to reduce any risk of bias. Last, the included studies mainly focused on developing ML models, while presenting their generalizability, robustness, or clinical usefulness was uncommon. Future studies should aim to demonstrate that ML positively impacts the quality and efficiency of healthcare.

## Figures and Tables

**Table 1:** Evaluation of risk of bias in studies included (*n* = 168) using the QUADAS-2 tool.

Sr. No. [Citation]	Data Selection: Risk of Bias/Applicability Concerns	Index Test: Risk of Bias/Applicability Concerns	Reference Standard: Risk of Bias/Applicability Concerns	Flow and Timing: Risk of Bias
1. [12]	high/high	low/high	high/high	low
2. [13]	low/low	low/low	low/low	low
3. [14]	high/low	low/low	low/low	low
4. [15]	low/low	low/high	high/high	low
5. [16]	low/low	low/low	low/low	low
6. [17]	high/high	low/high	high/high	low
7. [18]	high/high	low/low	high/low	low
8. [19]	low/low	low/high	low/low	low
9. [20]	low/low	low/low	low/high	low
10. [21]	high/high	low/low	high/low	low
11. [22]	high/high	low/low	high/high	low
12. [23]	high/low	high/low	high/low	low
13. [24]	low/high	low/low	high/high	low
14. [25]	high/high	high/low	low/low	low
15. [26]	low/low	high/low	low/low	low
16. [27]	high/low	low/low	high/low	low
17. [28]	high/high	low/low	high/low	low
18. [29]	high/low	low/low	high/low	low
19. [30]	high/high	low/low	high/low	low
20. [31]	high/high	low/high	high/low	low
21. [32]	high/high	high/high	high/high	low
22. [33]	low/low	low/low	low/low	low
23. [34]	low/high	low/low	low/high	low
24. [35]	high/high	low/low	low/low	low
25. [36]	low/low	low/low	low/low	low
26. [37]	high/high	low/low	high/low	low
27. [38]	high/high	low/low	high/low	low
28. [39]	high/high	low/low	high/low	low
29. [40]	high/high	high/low	high/low	low
30. [41]	low/low	low/low	low/low	low
31. [42]	high/low	high/low	low/low	low
32. [43]	low/high	low/high	low/high	low
33. [44]	low/low	high/low	high/low	low
34. [45]	high/high	low/high	low/high	low
35. [46]	high/low	low/low	low/low	low
36. [47]	high/high	low/low	low/low	low
37. [48]	high/high	low/high	low/high	low
38. [49]	low/low	low/low	high/low	low
39. [50]	low/high	low/low	high/low	high
40. [51]	low/high	low/low	low/low	low
41. [52]	high/low	low/high	high/low	low
42. [53]	high/high	low/low	low/low	high
43. [54]	low/low	low/high	low/high	low
44. [55]	high/high	low/low	high/low	low
45. [56]	high/high	low/high	high/low	low
46. [57]	high/high	low/low	high/high	low
47. [58]	high/high	high/high	high/high	low
48. [59]	low/high	low/low	high/high	low
49. [60]	low/high	low/low	high/high	low
50. [61]	low/low	low/low	high/low	high
51. [62]	high/high	low/low	high/low	low
52. [63]	low/high	low/high	high/high	low
53. [64]	high/high	high/high	high/high	low
54. [65]	high/high	low/low	high/low	low
55. [66]	low/high	low/low	high/low	low
56. [67]	high/high	low/high	low/high	low
57. [68]	low/high	low/low	low/low	high
58. [69]	low/low	low/low	low/low	low
59. [70]	high/high	low/low	low/low	low
60. [71]	low/low	low/low	low/low	low
61. [72]	low/high	low/low	high/low	low
62. [73]	low/low	low/low	high/low	low
63. [74]	low/low	low/low	low/low	low
64. [75]	low/low	low/low	low/low	low
65. [76]	low/low	low/low	low/low	low
66. [77]	high/high	high/low	high/low	low
67. [78]	high/low	high/low	high/low	low
68. [79]	high/low	high/low	high/low	low
69. [80]	high/low	high/low	low/low	low
70. [81]	low/low	low/low	low/low	low
71. [82]	low/low	low/low	high/low	low
72. [83]	low/low	low/low	low/low	low
73. [84]	high/low	low/low	high/low	low
74. [85]	low/low	low/low	low/low	high
75. [86]	high/high	low/low	low/low	low
76. [87]	high/high	high/low	low/low	low
77. [88]	low/low	low/low	low/low	low
78. [89]	high/high	high/high	high/high	low
79. [90]	high/high	high/high	high/high	low
80. [91]	high/high	low/low	high/low	low
81. [92]	low/low	low/low	high/low	low
82. [93]	low/high	low/low	high/high	low
83. [94]	low/low	low/low	low/low	high
84. [95]	high/high	high/low	high/high	low
85. [96]	low/high	high/low	high/high	low
86. [97]	high/high	low/high	low/high	low
87. [98]	high/high	low/low	low/low	low
88. [99]	low/high	low/high	high/high	low
89. [100]	low/high	low/high	high/high	low
90. [101]	low/high	low/low	low/high	low
91. [102]	high/high	low/low	high/low	low
92. [103]	low/low	low/low	low/low	low
93. [4]	high/low	low/high	high/high	low
94. [104]	low/low	low/low	high/low	low
95. [105]	high/high	low/high	high/low	low
96. [106]	low/high	low/low	low/high	low
97. [107]	low/low	low/low	high/low	low
98. [108]	low/low	low/low	low/low	low
99. [109]	high/high	high/low	high/low	low
100. [110]	low/low	low/low	high/low	low
101. [111]	low/low	low/low	high/low	low
102. [112]	high/low	high/low	high/high	low
103. [113]	high/high	low/low	low/high	high
104. [3]	low/high	low/low	low/low	low
105. [114]	low/low	low/low	low/low	low
106. [115]	low/low	low/low	low/low	low
107. [116]	high/high	high/low	high/low	low
108. [117]	high/low	high/low	low/low	low
109. [118]	high/high	low/low	high/low	low
110. [119]	low/low	low/low	low/low	low
111. [120]	low/low	low/high	high/high	low
112. [121]	low/low	low/low	high/low	low
113. [122]	high/high	high/low	low/low	low
114. [123]	low/low	low/low	low/low	low
115. [124]	low/high	low/low	high/low	low
116. [125]	high/high	low/low	low/high	low
117. [126]	high/low	high/low	high/low	high
118. [127]	high/high	low/low	high/low	low
119. [128]	low/low	high/low	low/low	low
120. [129]	high/low	low/low	low/low	low
121. [130]	high/high	low/low	high/low	high
122. [131]	high/low	high/low	high/low	low
123. [132]	high/high	low/low	high/low	low
124. [133]	high/high	low/low	high/low	high
125. [134]	low/high	high/low	high/low	low
126. [135]	high/low	high/low	low/low	low
127. [136]	high/low	high/low	high/low	low
128. [137]	high/low	high/high	low/low	low
129. [138]	low/high	low/high	high/low	low
130. [139]	high/low	low/low	low/low	low
131. [140]	high/low	low/high	high/high	low
132. [141]	low/low	low/low	high/low	low
133. [142]	high/high	low/low	high/low	low
134. [143]	high/high	low/low	low/low	low
135. [144]	high/high	low/low	high/low	low
136. [145]	high/high	high/low	high/low	low
137. [146]	high/high	low/low	high/low	low
138. [147]	high/low	high/low	low/low	low
139. [148]	high/high	low/low	high/low	low
140. [149]	high/high	low/high	high/high	low
141. [150]	high/high	low/high	high/high	low
142. [151]	low/high	low/low	high/high	low
143. [152]	high/high	low/high	high/high	low
144. [153]	high/low	low/low	high/low	low
145. [154]	low/low	low/high	high/high	low
146. [155]	low/low	high/low	low/low	low
147. [156]	low/high	low/low	low/low	low
148. [157]	high/high	high/low	high/low	high
149. [158]	low/low	low/low	low/low	low
150. [159]	low/high	low/high	low/high	low
151. [160]	high/low	low/high	low/low	low
152. [161]	low/low	high/low	high/low	high
153. [162]	high/low	low/low	low/high	low
154. [163]	low/low	low/high	low/high	low
155. [164]	high/low	low/low	high/low	low
156. [165]	low/low	low/low	high/low	low
157. [166]	low/high	low/high	high/high	high
158. [167]	low/low	low/low	low/low	low
159. [168]	low/low	low/low	high/low	low
160. [169]	low/high	high/low	high/high	low
161. [170]	high/high	low/low	low/low	low
162. [171]	low/low	low/low	high/low	low
163. [172]	low/low	low/low	low/low	low
164. [173]	low/low	low/low	high/low	low
165. [174]	low/low	low/low	low/low	low
166. [175]	high/high	high/high	high/high	low
167. [176]	high/high	high/low	low/low	low
168. [177]	high/high	low/low	high/low	low

**Table 2:** Number of studies in each field of dentistry, stratified by type of machine learning task (*n* = 168).

	Classification Task	Object Detection Task	Semantic Segmentation Task	Instance Segmentation Task	Generation Task
*n*	85	22	37	19	5
Field of dentistry, *n* (%)					
Restorative dentistry and endodontics	13 (15%)	1 (4%)	9 (24%)	2 (11%)	0 (0%)
Oral medicine	19 (22%)	5 (23%)	1 (3%)	0 (0%)	0 (0%)
Oral radiology	3 (4%)	0 (0%)	2 (5%)	2 (11%)	4 (80%)
Orthodontics	10 (12%)	3 (14%)	1 (3%)	3 (15%)	1 (20%)
Oral surgery and implantology	11 (13%)	3 (14%)	3 (8%)	0 (0%)	0 (0%)
Periodontology	9 (11%)	2 (9%)	7 (19%)	1 (5%)	0 (0%)
Prosthodontics	2 (2%)	0 (0%)	0 (0%)	0 (0%)	0 (0%)
Others (non-specific field, general dentistry)	18 (21%)	8 (36%)	14 (38%)	11 (58%)	0 (0%)

## Data Availability

All relevant data are available through the paper and supplementary material. Additional information is available from the authors upon reasonable request.

## Supplementary Materials

The following supporting information can be downloaded at: https://www.mdpi.com/article/10.3390/jcm12030937/s1, Search strategy, Figure S1: Geographical trends in number of publications of machine learning methods in dentistry between 1 January 2015 and 31 May 2021; Table S1: Studies included in the scoping review along with their characteristics (*n* = 168); Table S2: Studies excluded from the scoping review along with the reason for exclusion (*n* = 15); Table S3: Number of studies in each field of dentistry, stratified by the machine learning model used (*n* = 168); Table S4: Number of studies using the various performance metrics stratified by type of machine learning task. References [1,184,185,186,187,188,189,190,191,192,193,194,195,196,197] are cited in the Supplementary Materials.

## References

[1] Sun M.-L., Liu Y., Liu G.-M., Cui D., Heidari A.A., Jia W.-Y., Ji X., Chen H.-L., Luo Y.-G. (2020). Application of Machine Learning to Stomatology: A Comprehensive Review. IEEE Access.

[2] Schwendicke F., Chaurasia A., Arsiwala L., Lee J.-H., Elhennawy K., Jost-Brinkmann P.-G., Demarco F., Krois J. (2021). Deep learning for cephalometric landmark detection: Systematic review and meta-analysis. Clin. Oral Investig..

[3] Farhadian M., Shokouhi P., Torkzaban P. (2020). A decision support system based on support vector machine for diagnosis of periodontal disease. BMC Res. Notes.

[4] Abdalla-Aslan R., Yeshua T., Kabla D., Leichter I., Nadler C. (2020). An artificial intelligence system using machine-learning for automatic detection and classification of dental restorations in panoramic radiography. Oral Surg. Oral Med. Oral Pathol. Oral Radiol..

[5] Ben Li B., Feridooni T., Cuen-Ojeda C., Kishibe T., de Mestral C., Mamdani M., Al-Omran M. (2022). Machine learning in vascular surgery: A systematic review and critical appraisal. NPJ Digit. Med..

[6] Schwendicke F., Tzschoppe M., Paris S. (2015). Radiographic caries detection: A systematic review and meta-analysis. J. Dent..

[7] Moher D., Liberati A., Tetzlaff J., Altman D.G., PRISMA Group (2009). Preferred reporting items for systematic reviews and meta-analyses: The PRISMA statement. PLoS Med..

[8] Page M.J., McKenzie J.E., Bossuyt P.M., Boutron I., Hoffmann T.C., Mulrow C.D., Shamseer L., Tetzlaff J.M., Akl E.A., Brennan S.E. (2021). The PRISMA 2020 Statement: An Updated Guideline for Reporting Systematic Reviews. BMJ.

[9] Whiting P.F., Rutjes A.W.S., Westwood M.E., Mallett S., Deeks J.J., Reitsma J.B., Leeflang M.M.G., Sterne J.A.C., Bossuyt P.M.M., QUADAS-2 Group (2011). QUADAS-2: A Revised Tool for the Quality Assessment of Diagnostic Accuracy Studies. Ann. Intern. Med..

[10] Wolff R.F., Moons K.G., Riley R., Whiting P.F., Westwood M., Collins G.S., Reitsma J.B., Kleijnen J., Mallett S., for the PROBAST Group (2019). PROBAST: A Tool to Assess the Risk of Bias and Applicability of Prediction Model Studies. Ann. Intern. Med..

[11] Collins G.S., Reitsma J.B., Altman D.G., Moons K.G.M. (2015). Transparent reporting of a multivariable prediction model for individual prognosis or diagnosis (TRIPOD): The TRIPOD Statement. BMC Med..

[12] Aliaga I.J., Vera V., De Paz J.F., García A.E., Mohamad M.S. (2015). Modelling the Longevity of Dental Restorations by means of a CBR System. BioMed Res. Int..

[13] Gupta A., Kharbanda O.P., Sardana V., Balachandran R., Sardana H.K. (2015). A knowledge-based algorithm for automatic detection of cephalometric landmarks on CBCT images. Int. J. Comput. Assist. Radiol. Surg..

[14] Gupta A., Kharbanda O.P., Sardana V., Balachandran R., Sardana H.K. (2016). Accuracy of 3D cephalometric measurements based on an automatic knowledge-based landmark detection algorithm. Int. J. Comput. Assist. Radiol. Surg..

[15] Hadley A.J., Krival K.R., Ridgel A.L., Hahn E.C., Tyler D.J. (2015). Neural Network Pattern Recognition of Lingual–Palatal Pressure for Automated Detection of Swallow. Dysphagia.

[16] Kavitha M.S., An S.-Y., An C.-H., Huh K.-H., Yi W.-J., Heo M.-S., Lee S.-S., Choi S.-C. (2015). Texture analysis of mandibular cortical bone on digital dental panoramic radiographs for the diagnosis of osteoporosis in Korean women. Oral Surg. Oral Med. Oral Pathol. Oral Radiol..

[17] Mansoor A., Patsekin V., Scherl D., Robinson J.P., Rajwa B. (2015). A Statistical Modeling Approach to Computer-Aided Quantification of Dental Biofilm. IEEE J. Biomed. Health Inform..

[18] Jung S.-K., Kim T.-W. (2016). New approach for the diagnosis of extractions with neural network machine learning. Am. J. Orthod. Dentofac. Orthop..

[19] Kavitha M.S., Kumar P.G., Park S.-Y., Huh K.-H., Heo M.-S., Kurita T., Asano A., An S.-Y., Chien S.-I. (2016). Automatic detection of osteoporosis based on hybrid genetic swarm fuzzy classifier approaches. Dentomaxillofac. Radiol..

[20] Mahmoud Y.E., Labib S.S., Mokhtar H.M.O. Teeth periapical lesion prediction using machine learning techniques. Proceedings of the 2016 SAI Computing Conference (SAI).

[21] Wang L., Li S., Chen R., Liu S.-Y., Chen J.-C. (2016). An Automatic Segmentation and Classification Framework Based on PCNN Model for Single Tooth in MicroCT Images. PLoS ONE.

[22] De Tobel J., Radesh P., Vandermeulen D., Thevissen P.W. (2017). An automated technique to stage lower third molar development on panoramic radiographs for age estimation: A pilot study. J. Forensic Odonto-Stomatol..

[23] Hwang J.J., Lee J.-H., Han S.-S., Kim Y.H., Jeong H.-G., Choi Y.J., Park W. (2017). Strut analysis for osteoporosis detection model using dental panoramic radiography. Dentomaxillofac. Radiol..

[24] Imangaliyev S., van der Veen M.H., Volgenant C., Loos B.G., Keijser B.J., Crielaard W., Levin E. (2017). Classification of quantitative light-induced fluorescence images using convolutional neural network. arXiv.

[25] Johari M., Esmaeili F., Andalib A., Garjani S., Saberkari H. (2017). Detection of vertical root fractures in intact and endodontically treated premolar teeth by designing a probabilistic neural network: An ex vivo study. Dentomaxillofac. Radiol..

[26] Liu Y., Li Y., Fu Y., Liu T., Liu X., Zhang X., Fu J., Guan X., Chen T., Chen X. (2017). Quantitative prediction of oral cancer risk in patients with oral leukoplakia. Oncotarget.

[27] Miki Y., Muramatsu C., Hayashi T., Zhou X., Hara T., Katsumata A., Fujita H. (2017). Classification of teeth in cone-beam CT using deep convolutional neural network. Comput. Biol. Med..

[28] Oktay A.B. Tooth detection with Convolutional Neural Networks. Proceedings of the 2017 Medical Technologies National Congress (TIPTEKNO).

[29] Prajapati A.S., Nagaraj R., Mitra S. Classification of dental diseases using CNN and transfer learning. Proceedings of the 2017 5th International Symposium on Computational and Business Intelligence (ISCBI).

[30] Raith S., Vogel E.P., Anees N., Keul C., Güth J.-F., Edelhoff D., Fischer H. (2017). Artificial Neural Networks as a powerful numerical tool to classify specific features of a tooth based on 3D scan data. Comput. Biol. Med..

[31] Rana A., Yauney G., Wong L.C., Gupta O., Muftu A., Shah P. Automated segmentation of gingival diseases from oral images. Proceedings of the 2017 IEEE Healthcare Innovations and Point of Care Technologies (HI-POCT).

[32] Srivastava M.M., Kumar P., Pradhan L., Varadarajan S. (2017). Detection of tooth caries in bitewing radiographs using deep learning. arXiv.

[33] Štepanovský M., Ibrová A., Buk Z., Velemínská J. (2017). Novel age estimation model based on development of permanent teeth compared with classical approach and other modern data mining methods. Forensic Sci. Int..

[34] Yilmaz E., Kayikcioglu T., Kayipmaz S. (2017). Computer-aided diagnosis of periapical cyst and keratocystic odontogenic tumor on cone beam computed tomography. Comput. Methods Programs Biomed..

[35] Du X., Chen Y., Zhao J., Xi Y. A Convolutional Neural Network Based Auto-Positioning Method for Dental Arch In Rotational Panoramic Radiography. Proceedings of the 40th Annual International Conference of the IEEE Engineering in Medicine and Biology Society (EMBC).

[36] Egger J., Pfarrkirchner B., Gsaxner C., Lindner L., Schmalstieg D., Wallner J. Fully Convolutional Mandible Segmentation on a valid Ground- Truth Dataset. Proceedings of the 40th Annual International Conference of the IEEE Engineering in Medicine and Biology Society (EMBC).

[37] Fakhriy N.A.A., Ardiyanto I., Nugroho H.A., Pratama G.N.P. Machine Learning Algorithms for Classifying Abscessed and Impacted Tooth: Comparison Study. Proceedings of the 2018 2nd International Conference on Biomedical Engineering (IBIOMED).

[38] Fariza A., Arifin A.Z., Astuti E.R. Interactive Segmentation of Conditional Spatial FCM with Gaussian Kernel-Based for Panoramic Radiography. Proceedings of the 2018 International Symposium on Advanced Intelligent Informatics (SAIN).

[39] Gavinho L.G., Araujo S.A., Bussadori S.K., Silva J.V.P., Deana A.M. (2018). Detection of white spot lesions by segmenting laser speckle images using computer vision methods. Lasers Med. Sci..

[40] Ha S.-R., Park H.S., Kim E.-H., Kim H.-K., Yang J.-Y., Heo J., Yeo I.-S.L. (2018). A pilot study using machine learning methods about factors influencing prognosis of dental implants. J. Adv. Prosthodont..

[41] Heinrich A., Güttler F., Wendt S., Schenkl S., Hubig M., Wagner R., Mall G., Teichgräber U. (2018). Forensic Odontology: Automatic Identification of Persons Comparing Antemortem and Postmortem Panoramic Radiographs Using Computer Vision. Rofo.

[42] Jader G., Fontineli J., Ruiz M., Abdalla K., Pithon M., Oliveira L. Deep Instance Segmentation of Teeth in Panoramic X-ray Images. Proceedings of the 2018 31st SIBGRAPI Conference on Graphics, Patterns and Images (SIBGRAPI).

[43] Jiang M.-X., Chen Y.-M., Huang W.-H., Huang P.-H., Tsai Y.-H., Huang Y.-H., Chiang C.-K. Teeth-Brushing Recognition Based on Deep Learning. Proceedings of the 2018 IEEE International Conference on Consumer Electronics-Taiwan (ICCE-TW).

[44] Kim D.W., Kim H., Nam W., Kim H.J., Cha I.-H. (2018). Machine learning to predict the occurrence of bisphosphonate-related osteonecrosis of the jaw associated with dental extraction: A preliminary report. Bone.

[45] Lee J.-H., Kim D.-H., Jeong S.-N., Choi S.-H. (2018). Diagnosis and prediction of periodontally compromised teeth using a deep learning-based convolutional neural network algorithm. J. Periodontal Implant. Sci..

[46] Lee J.-H., Kim D.-H., Jeong S.-N., Choi S.-H. (2018). Detection and diagnosis of dental caries using a deep learning-based convolutional neural network algorithm. J. Dent..

[47] Lu S., Yang J., Wang W., Li Z., Lu Z. Teeth Classification Based on Extreme Learning Machine. Proceedings of the 2018 Second World Conference on Smart Trends in Systems, Security and Sustainability (WorldS4).

[48] Shah H., Hernandez P., Budin F., Chittajallu D., Vimort J.B., Walters R., Mol A., Khan A., Paniagua B. (2018). Automatic quantification framework to detect cracks in teeth. Proc. SPIE Int. Soc. Opt. Eng..

[49] Song B., Sunny S., Uthoff R., Patrick S., Suresh A., Kolur T., Keerthi G., Anbarani A., Wilder-Smith P., Kuriakose M.A. (2018). Automatic classification of dual-modalilty, smartphone-based oral dysplasia and malignancy images using deep learning. Biomed. Opt. Express.

[50] Thanathornwong B. (2018). Bayesian-Based Decision Support System for Assessing the Needs for Orthodontic Treatment. Health Inform. Res..

[51] Yang J., Xie Y., Liu L., Xia B., Cao Z., Guo C. Automated Dental Image Analysis by Deep Learning on Small Dataset. Proceedings of the 2018 IEEE 42nd Annual Computer Software and Applications Conference (COMPSAC).

[52] Yoon S., Odlum M., Lee Y., Choi T., Kronish I.M., Davidson K.W., Finkelstein J. (2018). Applying Deep Learning to Understand Predictors of Tooth Mobility Among Urban Latinos. Stud. Health Technol. Inform..

[53] Zakirov A., Ezhov M., Gusarev M., Alexandrovsky V., Shumilov E. (2018). Dental pathology detection in 3D cone-beam CT. arXiv.

[54] Zanella-Calzada L.A., Galván-Tejada C.E., Chávez-Lamas N.M., Rivas-Gutierrez J., Magallanes-Quintanar R., Celaya-Padilla J.M., Galván-Tejada J.I., Gamboa-Rosales H. (2018). Deep Artificial Neural Networks for the Diagnostic of Caries Using Socioeconomic and Nutritional Features as Determinants: Data from NHANES 2013–2014. Bioengineering.

[55] Zhang K., Wu J., Chen H., Lyu P. (2018). An effective teeth recognition method using label tree with cascade network structure. Comput. Med. Imaging Graph..

[56] Ali H., Khursheed M., Fatima S.K., Shuja S.M., Noor S. Object Recognition for Dental Instruments Using SSD-MobileNet. Proceedings of the 2019 International Conference on Information Science and Communication Technology (ICISCT).

[57] Alkaabi S., Yussof S., Al-Mulla S. Evaluation of Convolutional Neural Network based on Dental Images for Age Estimation. Proceedings of the 2019 International Conference on Electrical and Computing Technologies and Applications (ICECTA).

[58] Askarian B., Tabei F., Tipton G.A., Chong J.W. Smartphone-Based Method for Detecting Periodontal Disease. Proceedings of the 2019 IEEE Healthcare Innovations and Point of Care Technologies, (HI-POCT).

[59] Bouchahma M., Ben Hammouda S., Kouki S., Alshemaili M., Samara K. An Automatic Dental Decay Treatment Prediction using a Deep Convolutional Neural Network on X-ray Images. Proceedings of the 2019 IEEE/ACS 16th International Conference on Computer Systems and Applications (AICCSA).

[60] Casalegno F., Newton T., Daher R., Abdelaziz M., Lodi-Rizzini A., Schürmann F., Krejci I., Markram H. (2019). Caries Detection with Near-Infrared Transillumination Using Deep Learning. J. Dent. Res..

[61] Chen H., Zhang K., Lyu P., Li H., Zhang L., Wu J., Lee C.-H. (2019). A deep learning approach to automatic teeth detection and numbering based on object detection in dental periapical films. Sci. Rep..

[62] Cheng B., Wang W. (2019). Dental hard tissue morphological segmentation with sparse representation-based classifier. Med. Biol. Eng. Comput..

[63] Chin C.-L., Lin J.-W., Wei C.-S., Hsu M.-C. Dentition Labeling and Root Canal Recognition Using Ganand Rule-Based System. Proceedings of the 2019 International Conference on Technologies and Applications of Artificial Intelligence (TAAI).

[64] Choi H.-I., Jung S.-K., Baek S.-H., Lim W.H., Ahn S.-J., Yang I.-H., Kim T.-W. (2019). Artificial Intelligent Model with Neural Network Machine Learning for the Diagnosis of Orthognathic Surgery. J. Craniofac. Surg..

[65] Cui Z., Li C., Wang W. ToothNet: Automatic Tooth Instance Segmentation and Identification from Cone Beam CT Images. Proceedings of the 2019 IEEE/CVF Conference on Computer Vision and Pattern Recognition (CVPR).

[66] Dasanayaka C., Dharmasena B., Bandara W.R., Dissanayake M.B., Jayasinghe R. Segmentation of Mental Foramen in Dental Panoramic Tomography using Deep Learning. Proceedings of the 2019 14th Conference on Industrial and Information Systems (ICIIS).

[67] Cruz J.C.D., Garcia R.G., Cueto J.C.C.V., Pante S.C., Toral C.G.V. Automated Human Identification through Dental Image Enhancement and Analysis. Proceedings of the 2019 IEEE 11th International Conference on Humanoid, Nanotechnology, Information Technology, Communication and Control, Environment, and Management (HNICEM).

[68] Duong D.Q., Nguyen K.-C.T., Kaipatur N.R., Lou E.H.M., Noga M., Major P.W., Punithakumar K., Le L.H. Fully Automated Segmentation of Alveolar Bone Using Deep Convolutional Neural Networks from Intraoral Ultrasound Images. Proceedings of the 41st Annual International Conference of the IEEE Engineering in Medicine and Biology Society (EMBC).

[69] Ekert T., Krois J., Meinhold L., Elhennawy K., Emara R., Golla T., Schwendicke F. (2019). Deep Learning for the Radiographic Detection of Apical Lesions. J. Endod..

[70] Hatvani J., Basarab A., Tourneret J.-Y., Gyongy M., Kouame D. (2019). A Tensor Factorization Method for 3-D Super Resolution with Application to Dental CT. IEEE Trans. Med. Imaging.

[71] Hatvani J., Horvath A., Michetti J., Basarab A., Kouame D., Gyongy M. (2019). Deep Learning-Based Super-Resolution Applied to Dental Computed Tomography. IEEE Trans. Radiat. Plasma Med. Sci..

[72] Hegazy M.A.A., Cho M.H., Cho M.H., Lee S.Y. (2019). U-net based metal segmentation on projection domain for metal artifact reduction in dental CT. Biomed. Eng. Lett..

[73] Hiraiwa T., Ariji Y., Fukuda M., Kise Y., Nakata K., Katsumata A., Fujita H., Ariji E. (2019). A deep-learning artificial intelligence system for assessment of root morphology of the mandibular first molar on panoramic radiography. Dentomaxillofac. Radiol..

[74] Hu Z., Jiang C., Sun F., Zhang Q., Ge Y., Yang Y., Liu X., Zheng H., Liang D. (2019). Artifact correction in low-dose dental CT imaging using Wasserstein generative adversarial networks. Med. Phys..

[75] Hung M., Voss M.W., Rosales M.N., Li W., Su W., Xu J., Bounsanga J., Ruiz-Negrón B., Lauren E., Licari F.W. (2019). Application of machine learning for diagnostic prediction of root caries. Gerodontology.

[76] Ilic I., Vodanovic M., Subasic M. Gender Estimation from Panoramic Dental X-ray Images using Deep Convolutional Networks. Proceedings of the IEEE EUROCON 2019 -18th International Conference on Smart Technologies.

[77] Kats L., Vered M., Zlotogorski-Hurvitz A., Harpaz I. (2019). Atherosclerotic carotid plaque on panoramic radiographs: Neural network detection. Int. J. Comput. Dent..

[78] Kats L., Vered M., Zlotogorski-Hurvitz A., Harpaz I. (2018). Atherosclerotic carotid plaques on panoramic imaging: An automatic detection using deep learning with small dataset. arXiv.

[79] Kim D.W., Lee S., Kwon S., Nam W., Cha I.-H., Kim H.J. (2019). Deep learning-based survival prediction of oral cancer patients. Sci. Rep..

[80] Kim J., Lee H.-S., Song I.-S., Jung K.-H. (2019). DeNTNet: Deep Neural Transfer Network for the detection of periodontal bone loss using panoramic dental radiographs. Sci. Rep..

[81] Kise Y., Shimizu M., Ikeda H., Fujii T., Kuwada C., Nishiyama M., Funakoshi T., Ariji Y., Fujita H., Katsumata A. (2020). Usefulness of a deep learning system for diagnosing Sjögren’s syndrome using ultrasonography images. Dentomaxillofac. Radiol..

[82] Koch T.L., Perslev M., Igel C., Brandt S.S. Accurate segmentation of dental panoramic radiographs with U-NETS. Proceedings of the 2019 IEEE 16th International Symposium on Biomedical Imaging (ISBI 2019).

[83] Krois J., Ekert T., Meinhold L., Golla T., Kharbot B., Wittemeier A., Dörfer C., Schwendicke F. (2019). Deep Learning for the Radiographic Detection of Periodontal Bone Loss. Sci. Rep..

[84] Lee J.-S., Adhikari S., Liu L., Jeong H.-G., Kim H., Yoon S.-J. (2019). Osteoporosis detection in panoramic radiographs using a deep convolutional neural network-based computer-assisted diagnosis system: A preliminary study. Dentomaxillofac. Radiol..

[85] Li X., Zhang Y., Cui Q., Yi X., Zhang Y. (2019). Tooth-Marked Tongue Recognition Using Multiple Instance Learning and CNN Features. IEEE Trans. Cybern..

[86] Liu L., Xu J., Huan Y., Zou Z., Yeh S.-C., Zheng L.-R. (2020). A Smart Dental Health-IoT Platform Based on Intelligent Hardware, Deep Learning, and Mobile Terminal. IEEE J. Biomed. Health Inform..

[87] Liu Y., Shang X., Shen Z., Hu B., Wang Z., Xiong G. 3D Deep Learning for 3D Printing of Tooth Model. Proceedings of the 2019 IEEE International Conference on Service Operations and Logistics, and Informatics (SOLI).

[88] Milosevic D., Vodanovic M., Galic I., Subasic M. Estimating Biological Gender from Panoramic Dental X-ray Images. Proceedings of the 2019 11th International Symposium on Image and Signal Processing and Analysis (ISPA).

[89] Minnema J., van Eijnatten M., Hendriksen A.A., Liberton N., Pelt D.M., Batenburg K.J., Forouzanfar T., Wolff J. (2019). Segmentation of dental cone-beam CT scans affected by metal artifacts using a mixed-scale dense convolutional neural network. Med. Phys..

[90] Moriyama Y., Lee C., Date S., Kashiwagi Y., Narukawa Y., Nozaki K., Murakami S. Evaluation of Dental Image Augmentation for the Severity Assessment of Periodontal Disease. Proceedings of the 2019 International Conference on Computational Science and Computational Intelligence (CSCI).

[91] Moutselos K., Berdouses E., Oulis C., Maglogiannis I. Recognizing Occlusal Caries in Dental Intraoral Images Using Deep Learning. Proceedings of the 41st Annual International Conference of the IEEE Engineering in Medicine and Biology Society (EMBC).

[92] Murata M., Ariji Y., Ohashi Y., Kawai T., Fukuda M., Funakoshi T., Kise Y., Nozawa M., Katsumata A., Fujita H. (2019). Deep-learning classification using convolutional neural network for evaluation of maxillary sinusitis on panoramic radiography. Oral Radiol..

[93] Patcas R., Bernini D., Volokitin A., Agustsson E., Rothe R., Timofte R. (2019). Applying artificial intelligence to assess the impact of orthognathic treatment on facial attractiveness and estimated age. Int. J. Oral Maxillofac. Surg..

[94] Patcas R., Timofte R., Volokitin A., Agustsson E., Eliades T., Eichenberger M., Bornstein M.M. (2019). Facial attractiveness of cleft patients: A direct comparison between artificial-intelligence-based scoring and conventional rater groups. Eur. J. Orthod..

[95] Sajad M., Shafi I., Ahmad J. Automatic Lesion Detection in Periapical X-rays. Proceedings of the 2019 International Conference on Electrical, Communication, and Computer Engineering (ICECCE).

[96] Senirkentli G.B., Sen S., Farsak O., Bostanci E. A Neural Expert System Based Dental Trauma Diagnosis Application. Proceedings of the 2019 Medical Technologies Congress (TIPTEKNO).

[97] Stark B., Samarah M. Ensemble and Deep Learning for Real-time Sensors Evaluation of algorithms for real-time sensors with application for detecting brushing location. Proceedings of the 2019 IEEE 5th International Conference on Computer and Communications (ICCC).

[98] Tian S., Dai N., Zhang B., Yuan F., Yu Q., Cheng X. (2019). Automatic Classification and Segmentation of Teeth on 3D Dental Model Using Hierarchical Deep Learning Networks. IEEE Access.

[99] Tuzoff D.V., Tuzova L.N., Bornstein M.M., Krasnov A.S., Kharchenko M.A., Nikolenko S.I., Sveshnikov M.M., Bednenko G.B. (2019). Tooth detection and numbering in panoramic radiographs using convolutional neural networks. Dentomaxillofac. Radiol..

[100] Vinayahalingam S., Xi T., Bergé S., Maal T., de Jong G. (2019). Automated detection of third molars and mandibular nerve by deep learning. Sci. Rep..

[101] Woo J., Xing F., Prince J.L., Stone M., Green J.R., Goldsmith T., Reese T.G., Wedeen V.J., El Fakhri G. (2019). Differentiating post-cancer from healthy tongue muscle coordination patterns during speech using deep learning. J. Acoust. Soc. Am..

[102] Xu X., Liu C., Zheng Y. (2019). 3D Tooth Segmentation and Labeling Using Deep Convolutional Neural Networks. IEEE Trans. Vis. Comput. Graph..

[103] Yamaguchi S., Lee C., Karaer O., Ban S., Mine A., Imazato S. (2019). Predicting the Debonding of CAD/CAM Composite Resin Crowns with AI. J. Dent. Res..

[104] Yauney G., Rana A., Wong L.C., Javia P., Muftu A., Shah P. Automated Process Incorporating Machine Learning Segmentation and Correlation of Oral Diseases with Systemic Health. Proceedings of the 2019 41st Annual International Conference of the IEEE Engineering in Medicine and Biology Society (EMBC).

[105] Alalharith D.M., Alharthi H.M., Alghamdi W.M., Alsenbel Y.M., Aslam N., Khan I.U., Shahin S.Y., Dianišková S., Alhareky M.S., Barouch K.K. (2020). A Deep Learning-Based Approach for the Detection of Early Signs of Gingivitis in Orthodontic Patients Using Faster Region-Based Convolutional Neural Networks. Int. J. Environ. Res. Public Health.

[106] Aliaga I., Vera V., Vera M., García E., Pedrera M., Pajares G. (2020). Automatic computation of mandibular indices in dental panoramic radiographs for early osteoporosis detection. Artif. Intell. Med..

[107] Banar N., Bertels J., Laurent F., Boedi R.M., De Tobel J., Thevissen P., Vandermeulen D. (2020). Towards fully automated third molar development staging in panoramic radiographs. Int. J. Leg. Med..

[108] Cantu A.G., Gehrung S., Krois J., Chaurasia A., Rossi J.G., Gaudin R., Elhennawy K., Schwendicke F. (2020). Detecting caries lesions of different radiographic extension on bitewings using deep learning. J. Dent..

[109] Chang H.-J., Lee S.-J., Yong T.-H., Shin N.-Y., Jang B.-G., Kim J.-E., Huh K.-H., Lee S.-S., Heo M.-S., Choi S.-C. (2020). Deep Learning Hybrid Method to Automatically Diagnose Periodontal Bone Loss and Stage Periodontitis. Sci. Rep..

[110] Chen S., Wang L., Li G., Wu T.-H., Diachina S., Tejera B., Kwon J.J., Lin F.-C., Lee Y.-T., Xu T. (2020). Machine Learning in Orthodontics: Introducing a 3D Auto-segmentation and Auto-landmark Finder of Cbct Images To Assess Maxillary Constriction in Unilateral Impacted Canine patients. Angle Orthod..

[111] Chen Y., Du H., Yun Z., Yang S., Dai Z., Zhong L., Feng Q., Yang W. (2020). Automatic Segmentation of Individual Tooth in Dental CBCT Images from Tooth Surface Map by a Multi-Task FCN. IEEE Access.

[112] Chung M., Lee M., Hong J., Park S., Lee J., Lee J., Yang I.-H., Lee J., Shin Y.-G. (2020). Pose-aware instance segmentation framework from cone beam CT images for tooth segmentation. Comput. Biol. Med..

[113] Endres M., Hillen F., Salloumis M., Sedaghat A., Niehues S., Quatela O., Hanken H., Smeets R., Beck-Broichsitter B., Rendenbach C. (2020). Development of a Deep Learning Algorithm for Periapical Disease Detection in Dental Radiographs. Diagnostics.

[114] Fan F., Ke W., Wu W., Tian X., Lyu T., Liu Y., Liao P., Dai X., Chen H., Deng Z. (2020). Automatic human identification from panoramic dental radiographs using the convolutional neural network. Forensic Sci. Int..

[115] Fujima N., Andreu-Arasa V.C., Meibom S.K., Mercier G.A., Salama A.R., Truong M.T., Sakai O. (2020). Deep learning analysis using FDG-PET to predict treatment outcome in patients with oral cavity squamous cell carcinoma. Eur. Radiol..

[116] Fukuda M., Ariji Y., Kise Y., Nozawa M., Kuwada C., Funakoshi T., Muramatsu C., Fujita H., Katsumata A., Ariji E. (2020). Comparison of 3 deep learning neural networks for classifying the relationship between the mandibular third molar and the mandibular canal on panoramic radiographs. Oral Surg. Oral Med. Oral Pathol. Oral Radiol..

[117] Fukuda M., Inamoto K., Shibata N., Ariji Y., Yanashita Y., Kutsuna S., Nakata K., Katsumata A., Fujita H., Ariji E. (2020). Evaluation of an artificial intelligence system for detecting vertical root fracture on panoramic radiography. Oral Radiol..

[118] Geetha V., Aprameya K.S., Hinduja D.M. (2020). Dental caries diagnosis in digital radiographs using back-propagation neural network. Health Inf. Sci. Syst..

[119] Hung M., Hon E.S., Ruiz-Negron B., Lauren E., Moffat R., Su W., Xu J., Park J., Prince D., Cheever J. (2020). Exploring the Intersection between Social Determinants of Health and Unmet Dental Care Needs Using Deep Learning. Int. J. Environ. Res. Public Health.

[120] Hung M., Li W., Hon E.S., Su S., Su W., He Y., Sheng X., Holubkov R., Lipsky M.S. (2020). Prediction of 30-Day Hospital Readmissions for All-Cause Dental Conditions using Machine Learning. Risk Manag. Health Policy.

[121] Jaskari J., Sahlsten J., Järnstedt J., Mehtonen H., Karhu K., Sundqvist O., Hietanen A., Varjonen V., Mattila V., Kaski K. (2020). Deep Learning Method for Mandibular Canal Segmentation in Dental Cone Beam Computed Tomography Volumes. Sci. Rep..

[122] Jeong S.H., Yun J.P., Yeom H.-G., Lim H.J., Lee J., Kim B.C. (2020). Deep learning based discrimination of soft tissue profiles requiring orthognathic surgery by facial photographs. Sci. Rep..

[123] Joshi S.V., Kanphade R.D. (2020). Deep Learning Based Person Authentication Using Hand Radiographs: A Forensic Approach. IEEE Access.

[124] Kats L., Vered M., Blumer S., Kats E. (2020). Neural Network Detection and Segmentation of Mental Foramen in Panoramic Imaging. J. Clin. Pediatr. Dent..

[125] Khan H.A., Haider M.A., Ansari H.A., Ishaq H., Kiyani A., Sohail K., Muhammad M., Khurram S.A. (2021). Automated feature detection in dental periapical radiographs by using deep learning. Oral Surg. Oral Med. Oral Pathol. Oral Radiol..

[126] Kim H., Shim E., Park J., Kim Y.-J., Lee U., Kim Y. (2020). Web-based fully automated cephalometric analysis by deep learning. Comput. Methods Programs Biomed..

[127] Kim I., Misra D., Rodriguez L., Gill M., Liberton D.K., Almpani K., Lee J.S., Antani S. Malocclusion Classification on 3D Cone-Beam CT Craniofacial Images Using Multi-Channel Deep Learning Models. Proceedings of the 2020 42nd Annual International Conference of the IEEE Engineering in Medicine & Biology Society (EMBC).

[128] Kim J.-E., Nam N.-E., Shim J.-S., Jung Y.-H., Cho B.-H., Hwang J.J. (2020). Transfer Learning via Deep Neural Networks for Implant Fixture System Classification Using Periapical Radiographs. J. Clin. Med..

[129] Kunz F., Stellzig-Eisenhauer A., Zeman F., Boldt J. (2020). Artificial intelligence in orthodontics: Evaluation of a fully automated cephalometric analysis using a customized convolutional neural network. J. Orofac. Orthop..

[130] Kuramoto N., Ichimura K., Jayatilake D., Shimokakimoto T., Hidaka K., Suzuki K. Deep Learning-Based Swallowing Monitor for Realtime Detection of Swallow Duration. Proceedings of the 2020 42nd Annual International Conference of the IEEE Engineering in Medicine & Biology Society (EMBC).

[131] Kuwada C., Ariji Y., Fukuda M., Kise Y., Fujita H., Katsumata A., Ariji E. (2020). Deep learning systems for detecting and classifying the presence of impacted supernumerary teeth in the maxillary incisor region on panoramic radiographs. Oral Surg. Oral Med. Oral Pathol. Oral Radiol..

[132] Kwak G.H., Kwak E.-J., Song J.M., Park H.R., Jung Y.-H., Cho B.-H., Hui P., Hwang J.J. (2020). Automatic mandibular canal detection using a deep convolutional neural network. Sci. Rep..

[133] Lee D., Park C., Lim Y., Cho H. (2020). A Metal Artifact Reduction Method Using a Fully Convolutional Network in the Sinogram and Image Domains for Dental Computed Tomography. J. Digit. Imaging.

[134] Lee J.-H., Han S.-S., Kim Y.H., Lee C., Kim I. (2020). Application of a fully deep convolutional neural network to the automation of tooth segmentation on panoramic radiographs. Oral Surg. Oral Med. Oral Pathol. Oral Radiol..

[135] Lee J.-H.D., Jeong S.-N. (2020). Efficacy of deep convolutional neural network algorithm for the identification and classification of dental implant systems, using panoramic and periapical radiographs: A pilot study. Medicine.

[136] Lee J.-H., Kim D.-H., Jeong S.-N. (2020). Diagnosis of cystic lesions using panoramic and cone beam computed tomographic images based on deep learning neural network. Oral Dis..

[137] Lee J.-H., Kim Y.-T., Lee J.-B., Jeong S.-N. (2020). A Performance Comparison between Automated Deep Learning and Dental Professionals in Classification of Dental Implant Systems from Dental Imaging: A Multi-Center Study. Diagnostics.

[138] Lee J.-H., Yu H.-J., Kim M.-J., Kim J.-W., Choi J. (2020). Automated cephalometric landmark detection with confidence regions using Bayesian convolutional neural networks. BMC Oral Health.

[139] Lee K.-S., Jung S.-K., Ryu J.-J., Shin S.-W., Choi J. (2020). Evaluation of Transfer Learning with Deep Convolutional Neural Networks for Screening Osteoporosis in Dental Panoramic Radiographs. J. Clin. Med..

[140] Lee S., Woo S., Yu J., Seo J., Lee J., Lee C. (2020). Automated CNN-Based Tooth Segmentation in Cone-Beam CT for Dental Implant Planning. IEEE Access.

[141] Li C., Zhang D., Chen S. Research about Tongue Image of Traditional Chinese Medicine(TCM) Based on Artificial Intelligence Technology. Proceedings of the 2020 IEEE 5th Information Technology and Mechatronics Engineering Conference (ITOEC).

[142] Li Q., Chen K., Han L., Zhuang Y., Li J., Lin J. (2020). Automatic tooth roots segmentation of cone beam computed tomography image sequences using U-net and RNN. J. X-ray Sci. Technol..

[143] Li S., Pang Z., Song W., Guo Y., You W., Hao A., Qin H. Low-Shot Learning of Automatic Dental Plaque Segmentation Based on Local-to-Global Feature Fusion. Proceedings of the 2020 IEEE 17th International Symposium on Biomedical Imaging (ISBI).

[144] Lian C., Wang L., Wu T.-H., Wang F., Yap P.-T., Ko C.-C., Shen D. (2020). Deep Multi-Scale Mesh Feature Learning for Automated Labeling of Raw Dental Surfaces From 3D Intraoral Scanners. IEEE Trans. Med. Imaging.

[145] Mahdi F.P., Motoki K., Kobashi S. (2020). Optimization technique combined with deep learning method for teeth recognition in dental panoramic radiographs. Sci. Rep..

[146] Mallishery S., Chhatpar P., Banga K.S., Shah T., Gupta P. (2020). The precision of case difficulty and referral decisions: An innovative automated approach. Clin. Oral Investig..

[147] Matsuda S., Miyamoto T., Yoshimura H., Hasegawa T. (2020). Personal identification with orthopantomography using simple convolutional neural networks: A preliminary study. Sci. Rep..

[148] Boedi R.M., Banar N., De Tobel J., Bertels J., Vandermeulen D., Thevissen P.W. (2020). Effect of Lower Third Molar Segmentations on Automated Tooth Development Staging using a Convolutional Neural Network. J. Forensic Sci..

[149] Ngoc V.T.N., Agwu A.C., Son L.H., Tuan T.M., Giap C.N., Thanh M.T.G., Duy H.B., Ngan T.T. (2020). The Combination of Adaptive Convolutional Neural Network and Bag of Visual Words in Automatic Diagnosis of Third Molar Complications on Dental X-ray Images. Diagnostics.

[150] Oh J.H., Pouryahya M., Iyer A., Apte A.P., Deasy J.O., Tannenbaum A. (2020). A novel kernel Wasserstein distance on Gaussian measures: An application of identifying dental artifacts in head and neck computed tomography. Comput. Biol. Med..

[151] Orhan K., Bayrakdar I.S., Ezhov M., Kravtsov A., Özyürek T. (2020). Evaluation of artificial intelligence for detecting periapical pathosis on cone-beam computed tomography scans. Int. Endod. J..

[152] Ren J., Fan H., Yang J., Ling H. Detection of Trabecular Landmarks for Osteoporosis Prescreening in Dental Panoramic Radiographs. Proceedings of the 2020 42nd Annual International Conference of the IEEE Engineering in Medicine & Biology Society (EMBC).

[153] Schwendicke F., Elhennawy K., Paris S., Friebertshäuser P., Krois J. (2020). Deep learning for caries lesion detection in near-infrared light transillumination images: A pilot study. J. Dent..

[154] Setzer F.C., Shi K.J., Zhang Z., Yan H., Yoon H., Mupparapu M., Li J. (2020). Artificial Intelligence for the Computer-aided Detection of Periapical Lesions in Cone-beam Computed Tomographic Images. J. Endod..

[155] Sukegawa S., Yoshii K., Hara T., Yamashita K., Nakano K., Yamamoto N., Nagatsuka H., Furuki Y. (2020). Deep Neural Networks for Dental Implant System Classification. Biomolecules.

[156] Sun D., Pei Y., Song G., Guo Y., Ma G., Xu T., Zha H. Tooth Segmentation and Labeling from Digital Dental Casts. Proceedings of the 2020 IEEE 17th International Symposium on Biomedical Imaging (ISBI).

[157] Takahashi T., Nozaki K., Gonda T., Ikebe K. (2021). A system for designing removable partial dentures using artificial intelligence. Part 1. Classification of partially edentulous arches using a convolutional neural network. J. Prosthodont. Res..

[158] Tang W., Gao Y., Liu L., Xia T., He L., Zhang S., Guo J., Li W., Xu Q. (2020). An Automatic Recognition of Tooth- Marked Tongue Based on Tongue Region Detection and Tongue Landmark Detection via Deep Learning. IEEE Access.

[159] Thanathornwong B., Suebnukarn S. (2020). Automatic detection of periodontal compromised teeth in digital panoramic radiographs using faster regional convolutional neural networks. Imaging Sci. Dent..

[160] Vila-Blanco N., Carreira M.J., Varas-Quintana P., Balsa-Castro C., Tomas I. (2020). Deep Neural Networks for Chronological Age Estimation From OPG Images. IEEE Trans. Med. Imaging.

[161] Vranckx M., Van Gerven A., Willems H., Vandemeulebroucke A., Leite A.F., Politis C., Jacobs R. (2020). Artificial Intelligence (AI)-Driven Molar Angulation Measurements to Predict Third Molar Eruption on Panoramic Radiographs. Int. J. Environ. Res. Public Health.

[162] Wang X., Liu J., Wu C., Liu J., Li Q., Chen Y., Wang X., Chen X., Pang X., Chang B. (2020). Artificial intelligence in tongue diagnosis: Using deep convolutional neural network for recognizing unhealthy tongue with tooth-mark. Comput. Struct. Biotechnol. J..

[163] Wang Y., Hays R.D., Marcus M., Maida C.A., Shen J., Xiong D., Coulter I.D., Lee S.Y., Spolsky V.W., Crall J.J. (2020). Developing Children’s Oral Health Assessment Toolkits Using Machine Learning Algorithm. JDR Clin. Transl. Res..

[164] Welch M.L., McIntosh C., Purdie T.G., Wee L., Traverso A., Dekker A., Haibe-Kains B., Jaffray D.A. (2020). Automatic classification of dental artifact status for efficient image veracity checks: Effects of image resolution and convolutional neural network depth. Phys. Med. Biol..

[165] Welch M.L., McIntosh C., Traverso A., Wee L., Purdie T.G., Dekker A., Haibe-Kains B., Jaffray D.A. (2020). External validation and transfer learning of convolutional neural networks for computed tomography dental artifact classification. Phys. Med. Biol..

[166] Welikala R.A., Remagnino P., Lim J.H., Chan C.S., Rajendran S., Kallarakkal T.G., Zain R.B., Jayasinghe R.D., Rimal J., Kerr A.R. (2020). Automated Detection and Classification of Oral Lesions Using Deep Learning for Early Detection of Oral Cancer. IEEE Access.

[167] Yang H., Jo E., Kim H.J., Cha I.-H., Jung Y.-S., Nam W., Kim J.-Y., Kim J.-K., Kim Y.H., Oh T.G. (2020). Deep Learning for Automated Detection of Cyst and Tumors of the Jaw in Panoramic Radiographs. J. Clin. Med..

[168] You W., Hao A., Li S., Wang Y., Xia B. (2020). Deep learning-based dental plaque detection on primary teeth: A comparison with clinical assessments. BMC Oral Health.

[169] Zhang X., Liang Y., Li W., Liu C., Gu D., Sun W., Miao L. (2022). Development and evaluation of deep learning for screening dental caries from oral photographs. Oral Dis..

[170] Zheng Z., Yan H., Setzer F.C., Shi K.J., Mupparapu M., Li J. (2021). Anatomically Constrained Deep Learning for Automating Dental CBCT Segmentation and Lesion Detection. IEEE Trans. Autom. Sci. Eng..

[171] Zhu G., Piao Z., Kim S.C. Tooth Detection and Segmentation with Mask R-CNN. Proceedings of the 2020 International Conference on Artificial Intelligence in Information and Communication (ICAIIC).

[172] Alkhadar H., Macluskey M., White S., Ellis I., Gardner A. (2021). Comparison of machine learning algorithms for the prediction of five-year survival in oral squamous cell carcinoma. J. Oral Pathol. Med..

[173] Leite A.F., Van Gerven A., Willems H., Beznik T., Lahoud P., Gaêta-Araujo H., Vranckx M., Jacobs R. (2021). Artificial intelligence-driven novel tool for tooth detection and segmentation on panoramic radiographs. Clin. Oral Investig..

[174] Machado R.A., De Oliveira Silva C., Martelli-Junior H., das Neves L.T., Coletta R.D. (2021). Machine learning in prediction of genetic risk of nonsyndromic oral clefts in the Brazilian population. Clin. Oral Investig..

[175] Muramatsu C., Morishita T., Takahashi R., Hayashi T., Nishiyama W., Ariji Y., Zhou X., Hara T., Katsumata A., Ariji E. (2021). Tooth detection and classification on panoramic radiographs for automatic dental chart filing: Improved classification by multi-sized input data. Oral Radiol..

[176] Schwendicke F., Rossi J., Göstemeyer G., Elhennawy K., Cantu A., Gaudin R., Chaurasia A., Gehrung S., Krois J. (2021). Cost-effectiveness of Artificial Intelligence for Proximal Caries Detection. J. Dent. Res..

[177] Yasa Y., Çelik Ö., Bayrakdar I.S., Pekince A., Orhan K., Akarsu S., Atasoy S., Bilgir E., Odabaş A., Aslan A.F. (2021). An artificial intelligence proposal to automatic teeth detection and numbering in dental bite-wing radiographs. Acta Odontol. Scand..

[178] Rischke R., Schneider L., Müller K., Samek W., Schwendicke F., Krois J. (2022). Federated Learning in Dentistry: Chances and Challenges. J. Dent. Res..

[179] ITU/WHO (2018). Focus Group on “Artificial Intelligence for Health”. https://www.itu.int/en/ITU-T/focusgroups/ai4h/Pages/default.aspx.

[180] Schwendicke F., Singh T., Lee J.-H., Gaudin R., Chaurasia A., Wiegand T., Uribe S., Krois J., on behalf of the IADR e-Oral Health Network, the ITU WHO Focus Group AI for Health (2021). Artificial intelligence in dental research: Checklist for authors, reviewers, readers. J. Dent..

[181] Lones M. (2021). How to avoid machine learning pitfalls: A guide for academic researchers. arXiv.

[182] Stevens L.M., Mortazavi B.J., Deo R.C., Curtis L., Kao D.P. (2020). Recommendations for Reporting Machine Learning Analyses in Clinical Research. Circ. Cardiovasc. Qual. Outcomes.

[183] Vermeire T., Brughmans D., Goethals S., de Oliveira R.M.B., Martens D. (2022). Explainable image classification with evidence counterfactual. Pattern Anal. Appl..

[184] Nguyen K., Duong D., Almeida F., Major P., Kaipatur N., Pham T., Lou E., Noga M., Punithakumar K., Le L. (2020). Alveolar Bone Segmentation in Intraoral Ultrasonographs with Machine Learning. J. Dent. Res..

[185] Min X., Haijin C. Research on Rapid Detection of Tooth Profile Parameters of the Clothing Wires Based on Image Processing. Proceedings of the 2020 IEEE International Conference on Artificial Intelligence and Computer Applications (ICAICA).

[186] Rasteau S., Sigaux N., Louvrier A., Bouletreau P. (2020). Three-dimensional acquisition technologies for facial soft tissues—Applications and prospects in orthognathic surgery. J. Stomatol. Oral Maxillofac. Surg..

[187] Dot G., Rafflenbeul F., Arbotto M., Gajny L., Rouch P., Schouman T. (2020). Accuracy and reliability of automatic three-dimensional cephalometric landmarking. Int. J. Oral Maxillofac. Surg..

[188] Kapralos V., Koutroulis A., Irinakis E., Kouros P., Lyroudia K., Pitas I., Mikrogeorgis G. (2020). Digital subtraction radiography in detection of vertical root fractures: Accuracy evaluation for root canal filling, fracture orientation and width variables. An ex-vivo study. Clin. Oral Investig..

[189] Tanaka R., Tanaka T., Yeung A.W.K., Taguchi A., Katsumata A., Bornstein M.M. (2020). Mandibular Radiomorphometric Indices and Tooth Loss as Predictors for the Risk of Osteoporosis using Panoramic Radiographs. Oral Health Prev. Dent..

[190] Laishram A., Thongam K. Detection and Classification of Dental Pathologies using Faster-RCNN in Orthopantomogram Radiography Image. Proceedings of the 7th International Conference on Signal Processing and Integrated Networks (SPIN).

[191] Rao G.K.L., Mokhtar N., Iskandar Y.H.P., Srinivasa A.C. Learning Orthodontic Cephalometry through Augmented Reality: A Conceptual Machine Learning Validation Approach. Proceedings of the 2018 International Conference on Electrical Engineering and Informatics (ICELTICs).

[192] Damiani G., Grossi E., Berti E., Conic R., Radhakrishna U., Pacifico A., Bragazzi N., Piccinno R., Linder D. (2020). Artificial neural networks allow response prediction in squamous cell carcinoma of the scalp treated with radiotherapy. J. Eur. Acad. Dermatol. Venereol..

[193] Yoon S., Choi T., Odlum M., Mitchell D.A., Kronish I.M., Davidson K.W., Finkelstein J. (2018). Machine Learning to Identify Behavioral Determinants of Oral Health in Inner City Older Hispanic Adults. Stud. Health Technol. Inform..

[194] Yatabe M., Prieto J.C., Styner M., Zhu H., Ruellas A.C., Paniagua B., Budin F., Benavides E., Shoukri B., Michoud L. (2019). 3D superimposition of craniofacial imaging—The utility of multicentre collaborations. Orthod. Craniofacial Res..

[195] Hung M., Lauren E., Hon E.S., Birmingham W.C., Xu J., Su S., Hon S.D., Park J., Dang P., Lipsky M.S. (2020). Social Network Analysis of COVID-19 Sentiments: Application of Artificial Intelligence. J. Med. Internet Res..

[196] Suhail Y., Upadhyay M., Chhibber A. (2020). Kshitiz Machine Learning for the Diagnosis of Orthodontic Extractions: A Computational Analysis Using Ensemble Learning. Bioengineering.

[197] Mehandru N., Hicks W.L., Singh A.K., Markiewicz M.R. (2020). Machine Learning for Identification of Craniomaxillofacial Radiographic Lesions. J. Oral Maxillofac. Surg..

