# Supplementary material for: Machine Learning in Dentistry: A Scoping Review
Source: J Clin Med. 2023 Jan 25;12(3):937. doi: 10.3390/jcm12030937 (PMC9918184; doi:10.3390/jcm12030937)
Supplement: Supplementary file 1 [file jcm-12-00937-s001.zip › Supplementary.pdf]

Machine Learning in Dentistry: A Systematic Critical Appraisal

Supplementary Materials

Search strategy

- **Figure S1.** Geographical trends in number of publications of machine learning methods in dentistry between 1st January 2015 and 31st May 2021.
- **Table S1.** Studies included in the systematic critical appraisal along with their characteristics (*n* = 168). (Table S1 is available as an Excel document).
- **Table S2.** Studies excluded from the systematic critical appraisal along with the reason for exclusion (*n* = 15)
- **Table S3.** Number of performance metrics used in the included studies stratified by type of machine learning task

Search strategy

We show the search strategy for database IEEE Xplore below:

(((“Document Title”:“deep learning” OR “artificial intelligence” OR “machine learning” OR “convolutional neural network”) OR (“Keywords”:“deep learning” OR “artificial intelligence” OR “machine learning” OR “convolutional neural network”)) AND ((“Document Title”: “dental” OR “teeth) OR (“Keywords”:“dental” OR “teeth))).

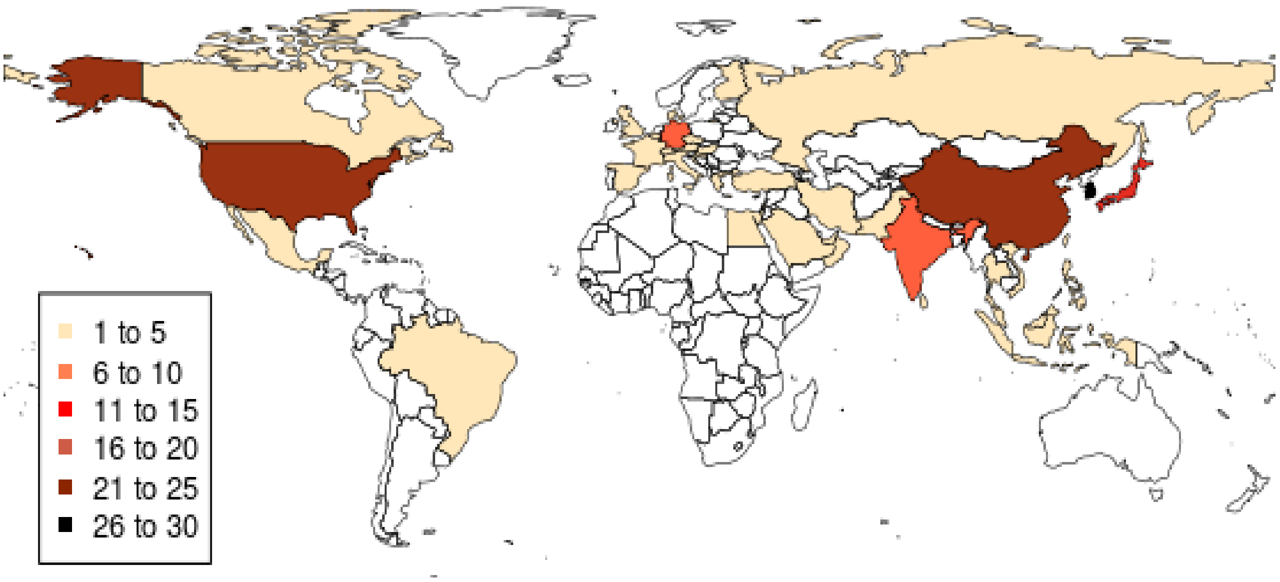

Figure S1. Geographical trends in number of publications of machine learning methods in dentistry between 1st January 2015 and 31st May 2021.

Table S2. Studies excluded from the systematic critical appraisal along with the reason for exclusion (n = 15).

	Citation	Reason for Exclusion from the Appraisal
1	Nguyen, K.; Duong, D.; Almeida, F.; Major, P.; Kaipatur, N.; Pham, T.; Lou, E.; Noga, M.; Punithakumar, K.; Le, L. Alveolar Bone Segmentation in Intraoral Ultrasonographs with Machine Learning. *J. Dent. Res.* **2020**, *99*, 1054–1061. https://doi.org/10.1177/0022034520920593.	Poor methodology/ reporting<br>• Reference test for the training and validation datasets was generated by only 1 professional expert, who was not formally trained in dentistry but was a biomedical engineer<br>• The validation set was also utilized during training to determine when to stop the parameter update to prevent overfitting
2	Sun, M.-L.; Liu, Y.; Liu, G.-M.; Cui, D.; Heidari, A.A.; Jia, W.-Y.; Ji, X.; Chen, H.-L.; Luo, Y.-G. Application of Machine Learning to Stomatology: A Comprehensive Review. *IEEE Access* **2020**, *8*, 184360–184374. https://doi.org/10.1109/access.2020.3028600.	A review article
3	Min, X.; Haijin, C. Research on Rapid Detection of Tooth Profile Parameters of the Clothing Wires Based on Image Processing. In Proceedings of the 2020 IEEE International Conference on Artificial Intelligence and Computer Applications (ICAICA), Dalian, China, 27–29 June 2020; pp. 586–590. https://doi.org/10.1109/icaica50127.2020.9182581.	Not an oral health topic
4	Rasteau, S.; Sigaux, N.; Louvrier, A.; Bouletreau, P. Three-dimensional acquisition technologies for facial soft tissues—Applications and prospects in orthognathic surgery. *J. Stomatol. Oral Maxillofac. Surg.* **2020**, *121*, 721–728. https://doi.org/10.1016/j.jormas.2020.05.013.	A review article
5	Dot, G.; Rafflenbeul, F.; Arbotto, M.; Gajny, L.; Rouch, P.; Schouman, T. Accuracy and reliability of automatic three-dimensional cephalometric landmarking. *Int. J. Oral Maxillofac. Surg.* **2020**, *49*, 1367–1378. https://doi.org/10.1016/j.ijom.2020.02.015.	A systematic review article
6	Kapralos, V.; Koutroulis, A.; Irinakis, E.; Kouros, P.; Lyroudia, K.; Pitas, I.; Mikrogeorgis, G. Digital subtraction radiography in detection of vertical root fractures: Accuracy evaluation for root canal filling, fracture orientation and width variables. An ex-vivo study. *Clin. Oral Investig.* **2020**, *24*, 3671–3681. https://doi.org/10.1007/s00784-020-03245-0.	No ML method used
7	Tanaka, R.; Tanaka, T.; Yeung, A.W.K.; Taguchi, A.; Katsumata, A.; Bornstein, M.M. Mandibular Radiomorphometric Indices and Tooth Loss as Predictors for the Risk of Osteoporosis using Panoramic Radiographs. *Oral Health Prev. Dent.* **2020**, *18*, 773–782. https://doi.org/10.3290/J.OHPD.A45081.	No ML method used
8	Laishram, A.; Thongam, K. Detection and Classification of Dental Pathologies using Faster-RCNN in Orthopantomogram Radiography Image. In Proceedings of the 7th International Conference on Signal Processing and Integrated Networks (SPIN), Noida, India, 27–28 February 2020; pp. 423–428. https://doi.org/10.1109/spin48934.2020.9071242.	Poor methodology/ reporting<br>• Labeled bounding boxes were generated by a software tool to serve as the reference test for the training dataset but were not checked for errors by a human expert<br>• Model architecture not adequately described, example, number of convolutional layers<br>• Some results are shown via images which have poor resolution<br>• Absence of the Discussion section of the paper. Hence placing the results in the context of the previous and current research is lacking.
9	Rao, G.K.L.; Mokhtar, N.; Iskandar, Y.H.P.; Srinivasa, A.C. Learning Orthodontic Cephalometry through Augmented Reality: A Conceptual Machine Learning Validation Approach. In Proceedings of the 2018 International Conference on Electrical Engineering and Informatics (ICELTICs), Banda Aceh, Indonesia, 19–20 September 2018; pp. 133–138. https://doi.org/10.1109/iceltics.2018.8548939.	A conceptual review article
10	Damiani, G.; Grossi, E.; Berti, E.; Conic, R.; Radhakrishna, U.; Pacifico, A.; Bragazzi, N.; Piccinno, R.; Linder, D. Artificial neural networks allow response prediction in squamous cell carcinoma of the scalp treated with radiotherapy. *J. Eur. Acad. Dermatol. Venereol.* **2020**, *34*, 1369–1373. https://doi.org/10.1111/jdv.16210.	Not an oral health topic
11	Yoon, S.; Choi, T.; Odlum, M.; Mitchell, D.A.; Kronish, I.M.; Davidson, K.W.; Finkelstein, J. Machine Learning to Identify Behavioral Determinants of Oral Health in Inner City Older Hispanic Adults. *Stud. Health Technol. Inform.* **2018**, *251*, 253–256. https://doi.org/10.3233/978-1-61499-880-8-253.	Poor methodology/ reporting<br>• The paper does not discuss how it's specific research question is tied to the larger context of oral health in USA<br>• The study used 6 deep neural network models for variable selection but no further details are given<br>• The study also used 10 data mining algorithms, whose names are listed but no further details are provided
12	Yatabe, M.; Prieto, J.C.; Styner, M.; Zhu, H.; Ruellas, A.C.; Paniagua, B.; Budin, F.; Benavides, E.; Shoukri, B.; Michoud, L.; et al. 3D superimposition of craniofacial imaging—The utility of multicentre collaborations. *Orthod. Craniofacial Res.* **2019**, *22* (Suppl. 1), 213–220. https://doi.org/10.1111/ocr.12281.	A supplement article (similar to a review article)
13	Hung, M.; Lauren, E.; Hon, E.S.; Birmingham, W.C.; Xu, J.; Su, S.; Hon, S.D.; Park, J.; Dang, P.; Lipsky, M.S. Social Network Analysis of COVID-19 Sentiments: Application of Artificial Intelligence. *J. Med. Internet Res.* **2020**, *22*, e22590. https://doi.org/10.2196/22590.	Not an oral health topic
14	Suhail, Y.; Upadhyay, M.; Chhibber, A.; Kshitiz Machine Learning for the Diagnosis of Orthodontic Extractions: A Computational Analysis Using Ensemble Learning. *Bioengineering* **2020**, *7*, 55. https://doi.org/10.3390/bioengineering7020055.	Poor methodology/ reporting<br>• The authors selected 19 feature variables or elements that characterize orthodontic problems and are assumed to be important in extraction decisions based on existing orthodontic literature. But these 19 variables or elements are not named or described further.<br>• Performance metrics, such as accuracy and error rate, were measured and reported via bar-charts but were not specified in the text. This hampered the evaluation of the results and their interpretation.

15 Mehandru, N.; Hicks, W.L.; Singh, A.K.; Markiewicz, M.R. Machine Learning for Identification of Craniomaxillofacial Radiographic Lesions. *J. Oral Maxillofac. Surg.* **2020**, *78*, 2106–2107. https://doi.org/10.1016/j.joms.2020.07.010. A review article

Table S3. Number of performance metrics used in the included studies stratified by type of machine learning task.

	Classification task	Object detection task	Semantic segmentation task	Instance segmentation task	Generation task
Number of studies	85	22	37	19	5
Performance metrics					
Accuracy	65	9	12	17	
Intersection over union or DICE indices or Jaccard similarity coefficient		4	26	9	2
Sensitivity or recall or true positve rate	55	19	22	11	
Precision or positive predictive value	30	12	15	7	
Mean average precision		2			
Area under the receiver-operating curve	36	3	4		
F indices	16	7	6	4	
Specificity or true negative rate	34	4	8	4	
Negative predictive value	8		3	1	
Rank-N recognition rate	1				
Mean or normalized absolute difference	3	2	2	3	1
Relative error or mean error rate or root mean squared error	8	2	4	4	3
Correlation coefficients (Intra-class or Matthew's or Pearson's correlation coefficient or Cohen's kappa)	10	1	4	4	
Confusion matrices	8	3	2	1	
Time taken for analysis	6	1	1	4	2
Co-efficient of variation		1	2		
Failure rate	2	1			
Intra-CNN, inter-CNN consistency values	1		1		
Area under the precision recall curve	3				
Youden index	1				
False positive rate	2	1	2		
Difference between volumes or surfaces or points (Hausdorff distance, Relative volume difference, Average symmetric surface distance, Mean curve distance)			6	3	1
Incremental cost-effectiveness ratio			1		
Structural similarity			3		3
Sum of square difference			1		
Peak signal-to-noise ratio					3

Note: Most studies reported multiple metrics.
